# Building community resilience in a context of climate change: The role of social capital

**DOI:** 10.1007/s13280-021-01678-9

**Published:** 2022-01-11

**Authors:** Esther Carmen, Ioan Fazey, Helen Ross, Melissa Bedinger, Fiona M. Smith, Katrin Prager, Kerri McClymont, David Morrison

**Affiliations:** 1 Department of Environment and Geography, University of York, Heslington, York, YO10 5NG UK; 2 School of Agriculture and Food Sciences, The University of Queensland, Brisbane, QLD 4072 Australia; 3 School of Energy, Geoscience Infrastructure and Society, Heriot Watt University, Edinburgh, UK; 4 School of Geosciences, University of Aberdeen, St Mary’s, Elphinstone Road, Aberdeen, AB24 3UF UK; 5 Geography, School of Social Sciences, University of Dundee, Perth Road, Dundee, DD14HN UK

**Keywords:** Community resilience building, Climate change, Social capital, Socio-cultural factors

## Abstract

Social capital is considered important for resilience across social levels, including communities, yet insights are scattered across disciplines. This meta-synthesis of 187 studies examines conceptual and empirical understandings of how social capital relates to resilience, identifying implications for community resilience and climate change practice. Different conceptualisations are highlighted, yet also limited focus on underlying dimensions of social capital and proactive types of resilience for engaging with the complex climate change challenge. Empirical insights show that structural and socio-cultural aspects of social capital, multiple other factors and formal actors are all important for shaping the role of social capital for guiding resilience outcomes. Thus, finding ways to work with these different elements is important. Greater attention on how and why outcomes emerge, interactions between factors, approaches of formal actors and different socio-cultural dimensions will advance understandings about how to nurture social capital for resilience in the context of climate change.

## Introduction

With growing recognition of the potential severity of human induced climate change (Goss et al. 2020; Steg 2018), interest in how local communities and diverse actors can become more resilient in the face of climate related shocks and stressors is rapidly growing (Brown 2014; Elmqvist et al. 2019). This growing interest on resilience^1^ building—broadly defined as the ability of place-based or interest based communities to proactively cohere and develop abilities to be able to adapt in the face of different kinds of shocks and stressors (Berkes and Ross 2013; Patel et al. 2017; Vaneeckhaute et al. 2017; Fazey et al. 2021)—has occurred alongside a rapidly growing body of research from diverse disciplines on resilience more generally and in relation to a diversity of contexts and fields of study (Matarrita-Cascante et al. 2017). Through such work a plethora of different factors that enhance or constrain resilience at community levels and other social levels^2^ have been identified (Urquiza et al. 2021; Umamaheswari et al. 2021). Such work highlights, for example, how enhancing community resilience requires approaches well beyond just technical or infrastructural interventions to including consideration of diverse social and psychological factors. For example, in the field of emergency management quality infrastructure (e.g. roads and housing) is an important factor to access vital services (e.g. food and health care) (Javadpoor et al. 2021). In other fields, such as rural development and urban studies, diverse incomes and institutions that mediate interests and access to resources and opportunities are important in shaping abilities to overcome shocks and to adapt to long-term stresses (Tajuddin and Dąbrowski 2021; Pandey et al. 2021).

A core subset of such research related to community resilience building, and often resilience more generally, has broadly focused on the role of different social factors that shape potential or actual collective action for change and resilience (Maclean et al. 2014). Such work has highlighted, for example, the importance of local knowledge sharing, clear communication, social learning and people–place connections which shape perceptions and actions in relation to increased risk of extreme weather events (Maclean et al. 2014; Bowser and Cutter 2015; Wilson et al. 2020); or the importance of safety nets and factors such as confidence and aspirations for overcoming threats to food security (Gambo Boukary et al. 2016; Smith and Frankenberger 2018).

Many of these studies referring to different social factors use the lens of social capital (e.g. Mngumi 2020). This conceptual lens focuses attention on the social relationships, networks and trust in shaping outcomes (Jordan 2015; MacGillivray 2018), such as in mobilising collective action within and between different social groups for disaster management, in general to respond to a range of shocks (Aldrich and Meyer 2015) and in terms of specific threats such as wildfires (Jacobs and Cramer 2017). Also social capital to promote and coordinate collective actions in communities to adapt to climate change (Adger 2003) and informally within organisations (Pelling et al. 2008), and for supporting community innovation for renewable energy initiatives for climate mitigation (Morrison and Ramsey 2019).

The literature on social capital and/or resilience is now vast and drawing out its implications for informing community resilience building conceptually and in practice is a significant challenge. While there have been past reviews (e.g. Aldrich and Meyer 2015; Rockenbauch and Sakdapolrak 2017), two important knowledge gaps remain. First, there has been limited focus on the practical insights emerging from studies of social capital and community resilience building for action-orientated knowledge on how to better navigate and work with the multiple factors (including social capital) to actively enhance resilience across community settings, with many reviews instead usually focusing on higher level conceptual foundations. Second, reviews have also tended to underplay the issue that the way both resilience and social capital are framed together has significant implications for the research being conducted and any conclusions being drawn from them. This has limited the possibility for more nuanced understandings of both the broader conceptual and practical insights and identification of the critical knowledge gaps that need to be addressed in future research.

This paper therefore aims to review research on the role of social capital in supporting resilience building to identify practical implications and knowledge gaps broadly within the context of climate change. We first provide a brief overview of both social capital and resilience and explain how the review was approached. We then present our findings on how social capital and/or resilience is being conceptualised and the empirical findings about how social capital can shape resilience. Finally, critical research gaps are identified to better understand how to apply social capital approaches to promote community resilience in relation to climate change.

## Resilience, social capital and climate change

This review seeks to understand the role of social capital in supporting community resilience building. Community resilience is has been defined as ‘the existence, development and engagement of community resources by community members to thrive in an environment characterised by change, uncertainty, unpredictability and surprise (Magis 2010, p. 402) or resilience ‘as a process linking a set of adaptive capacities to a positive trajectory of functioning and adaptation’ (Norris et al. 2008, p. 127). Emphasis has also been placed on the way human social aspects are closely intertwined with ecological dimensions and dynamics (Folke 2006) with much of what happens at community levels being influenced by interactions at multiple levels and scales (Holling 2001; Folke 2006). Importantly, and as highlighted by Ross and Berkes (2014), much of the understanding of resilience requires finding ways to combine different factors and influences to inform how community resilience building may be advanced, recognising that it is ultimately a process rather than necessarily an end goal that involves developing different forms of adaptive capacity and agency (Berkes and Ross 2013). Community resilience is thus often closely linked to different aspects of economic development (Sherrieb et al. 2010) and requires attention to normative aspects, and being driven by values and human agency that shape goals and how social action unfolds. For example, to enhance control of land by communities, land first needs to be collectively recognised as important, policy environments also need to shift to enhance community control in principle and different capacities and resources need to be actively brought together to bring this about in practice (Skerratt 2013).

A community perspective emphasises community actors developing and engaging resources for the community to thrive in the face of change (Magis 2010). Such change, for example, can be more specific and, while being unanticipated, easily identifiable such as fires or floods. Other change may be more diverse or unanticipated. Different kinds of resilience—generalised or specific—then require different approaches to resilience building (Jacobs and Cramer 2017). In the context of challenges such as climate change a narrow focus on specified resilience (e.g. of economic assets, to specific threats) may hinder more generalised community resilience (Folke et al. 2010). Thus some approaches to building resilience may have limited potential for engaging with the complexities of climate change (Pelling 2011; Urquiza et al. 2021). As such, there has been a growing interest in developing integrated, systemic approaches to enhance specified and generalised resilience concurrently (Berkes and Ross 2016) and to working with issues that may arise over longer time frames (Fazey et al. 2018) and which more effectively integrate normative dimensions such as values, social norms and power (Walsh-Dilley et al. 2016). While there are many different understandings of resilience, we broadly take the view that community resilience building requires both focus and holism, and thus requires attending to systemic aspects and working with different actors, perspectives, needs and diverse facets of the climate change challenge (Table 1).

**Table 1:** Key elements for community resilience and climate change practice [Adapted from Fazey et al. (2018)]

Developing and maintaining adaptability and flexibility to continue to guide and draw on different resources and capacities when needed
Accounting for shocks (e.g. floods) and stresses (e.g. food insecurity), direct and indirect foreseen and unforeseen changes and outcomes to build specific and generalised resilience
Including diverse perspectives by connecting horizontally (e.g. across social groups/ policy sectors) to develop novel synergistic solutions to address multiple concerns
Strengthening vertical connections across social levels (individual, family, community, government organisations), engaging with issues of social power to enhance support and enable collaborative action
Engaging in transformative action to proactively reduce carbon emissions
Drawing on positive climate narratives to create hope and inspire action
Fostering creativity and imagination to envisage alternative futures to guide change
Ensuring climate disadvantage and reducing inequities is a core dimension in decision-making to overcome injustices of climate change and climate action
Crafting processes and pathways by encouraging meaningful participation, learning and empowering for and through change
Creating transformative change, rather than adjusting or reforming existing conditions

Like resilience, social capital is another contested concept with many different interpretations. Social capital has been applied in many disciplines from sociology (Bourdieu 1986; Coleman 1988), political science (Putnam 1995), economics (Woolcock 1998); community and international development (Tenzin and Natsuda 2016). The concept has often been applied to highlight opportunities to enhance initiatives or programmes at diverse levels—such as for enhancing household food security through memberships of farmers’ organisations and involvement in other community activities (Dzanja et al. 2015), at the community level to maximise opportunities to enhance collective social capital within different types of interventions (e.g. participatory interventions) (Lang and Ramírez 2017), or for large-scale regions to ensure social capital is maintained in the immediate aftermath of flooding to strengthen the potential for long-term recovery (Akbar and Aldrich 2018).

In studies at community levels, social capital is often framed or defined as social relationships or networks and how this provides a utilitarian *resource* (e.g. for helping a household manage challenges and risks or to seize opportunities) (Habibov and Afand 2017). Such conceptualisations are broadly structural and focus on connections between actors, content-based approaches emphasising attributes that shape outcomes (e.g. norms of reciprocity, trust and shared goals), and approaches that aim to integrate these (Phillips 2016). Recognition that diverse types, qualities and magnitudes of outcomes emerge from social relationships has involved a shift away from framing social capital as being only positive (Portes 1998), such as with many examples of social capital reinforcing socio-economic exclusion and resistance (Wilshusen 2009; Adhikari and Goldey 2010) and which has led to an increasing emphasis on the importance of power and norms in shaping what unfolds and for whom from social capital (Gelderblom 2018).

Given that much of the emphasis on community resilience is often assumed to come from the way in which individuals and groups are able to organise (Grube and Storr 2014) and that the concept of social capital is often assumed to be a core mechanism underpinning effective individual and collective action (Adger 2003), it is then not surprising that there have been a large number of studies which, in some way have examined the interaction between the two. The concept of social capital, for example, has been widely used to understand interventions aiming to enhance adaptive capacities and resilience. This includes those directly related to climate change and the community level, such as in relation to natural hazards (Babcicky and Seebauer 2017) and more generally at the community level in terms of supporting health outcomes (Cattell 2001), enhancing economic development (Flora et al. 1997), and increasing participation in collective decision-making (Cleaver 2005). Social capital has also often been viewed conceptually as core for community resilience (Adger 2000; Berkes and Ross 2013).

In summary, building community resilience in the context of climate change is an important but complex social process (Fazey et al. 2018). It is inherently linked in diverse ways to social dynamics such as social relationships and networks that are often studied or understood through the lens of social capital. While there have been many different studies on the relationships between resilience building and social capital, there has been rather limited emphasis on reviews that draw out the implications of social capital for the practice of resilience building more generally. This has been hampered by the many different interpretations of both resilience and social capital. This review therefore seeks to draw out practical and empirical insights from different studies in a way that considers the different ways in which resilience and social capital have been understood.

## Methodology

This review was approached as a meta-synthesis employing interpretivist and qualitative methods to generate substantive and integrated findings (Finfgeld 2003; Zimmer 2006). In this process a modified version of more systematic review processes was used (Fazey et al. 2004). First, a wide range of peer reviewed articles were identified through search engines (e.g. Scopus) identifying papers by searching using both the terms resilience and social capital from titles, abstracts or key words. This resulted in 262 articles. Articles were excluded if they appeared in the search multiple times, were not published in English, or could not be accessed, reducing the set to 187 papers.

Qualitative and inductive methods were used for analysis. This included descriptive NVivo coding (Saldana 2016) to identify text in the articles relating to: (1) conceptualisations of social capital, resilience and the role of social capital; and (2) empirical findings, with care taken to avoid subjective and speculative discussion about the empirical findings (Bondas and Hall 2007). Codes were developed and applied iteratively across studies to allow for new interpretations and potential codes to emerge through the process (Strauss and Corbin 1994). Codes and their interconnections were then explored using visual mapping techniques to develop themes (Ritchie et al. 2003). A modified version of the pattern matching (Cao 2007) was also used to compare and contrast patterns (Trochim 1989).

It is important to note that this review was not exhaustive; rather it provides an indicative account of what the literature overall tells us about social capital and resilience building. There are thousands of papers on resilience and associated social issues, and many of these would broadly relate to the topic. Many pragmatic judgments (e.g. level of engagement with related concepts or understandings in studies orientated to specific contexts, such as migration) were needed to ensure the review was sufficiently focused while also encompassing of a diversity of studies. As is the case with many qualitative studies, the emphasis was therefore on identifying broad patterns by seeking diversity of different studies and interpretations, rather than trying to present a more quantified view of what was present in the literature as a whole. Finally, the included papers did not always relate directly to climate change or to the community level. Our goal was to bring together more generalised insights about the nature and role of social capital in resilience that could then be applied more broadly to community resilience building within a context of major challenges like climate change.

## Results and Discussion

### What ways have resilience, social capital and the role of social capital been conceptualised?

#### Concepts of resilience and social capital

Around three quarters of studies provided definitions of resilience. Among these, there were three general interpretations of resilience: (1) reactive resilience; (2) responsive resilience; or (3) proactive resilience. The vast majority of studies viewed resilience as reactive or responsive, with few (around one tenth) defining it as proactive.

Reactive resilience concerned actions to cope with the immediate aftermath of a shock, with an assumed goal of stability and a timely return to the status quo, i.e. to resume ‘business as usual’. This conceptualisation often assumed the need for top-down command and control (Murphy 2007) or unsupported actions undertaken by local people (Uekusa and Matthewman 2016).

In contrast, responsive resilience was viewed as learning from shocks, to enact adjustments to social, environmental or physical components, i.e. to strengthen the existing system to reduce negative consequences from future shocks. Here, resilience was viewed as multifaceted, encompassing different actors, interests and capacities (Vallance and Carlton 2015) as part of an ongoing process of change (Exner et al. 2016).

Finally, proactive resilience involved an ongoing process of foresight, experimentation, reflection and learning, requiring systemic perspectives and multi-scalar approaches involving norms, identities and values and potential need for radical change. This view highlighted the influence of governance arrangements, meanings and power dynamics and the importance of redundancy, flexibility and proactively working to shape complex, non-linear, dynamic and context specific change processes (Kizos et al. 2014).

Considering the multiple dynamic ways climate change interacts with multiple social levels, the climate challenge is not likely to be addressed without such system-oriented change that creates opportunities for alternative ways of thinking and acting (Pelling et al. 2015). Enhancing proactive resilience is therefore much more likely to be relevant than resilience types that emphasise maintenance of the status quo. Despite this, very few studies viewed resilience as a proactive process, with most conceptualising resilience as either reactive or responsive.

Turning to social capital, around three quarters of studies defined this explicitly. Four broad definitions of social capital were identified as: (1) social networks; (2) social networks and outcomes; (3) social networks, trust and norms of reciprocity; and (4) social networks and socio-cultural dimensions. Of the studies defining social capital, around a third defined social capital as social networks (1), with other definitions each accounting for around one fifth of studies.

Within this, two typologies of network connections were often used. These were: ‘strong/ weak ties’ or, more frequently, ‘bonding/ bridging/ linking social capital’. These differentiated along ideas of ‘homophily’ or ‘sameness’ and ‘heterogeneity’ or ‘difference’ between people and groups. These distinguish ‘bonding social capital’ and ‘strong ties’ for interpersonal relationships (Barrett et al. 2011), ‘bridging social capital’ or ‘weak tie’ across different social groups (Islam and Walkerden 2014); and/or ‘linking social capital’, emphasising connection across formal hierarchies, (e.g. between community and government actors) (Parés et al. 2018) which implicitly acknowledges underlying power differentials.

The first conceptualisation viewed social capital as social networks that connect people (Carpenter 2015), e.g. though membership of formal groups (Kim and Marcouiller 2016). The second included social networks and associated outcomes, e.g. improved health, information or civil engagement (Barrett et al. 2011; Cairns-Nagi and Bambra 2013). The third conceptualisation viewed social capital as social networks combined with trust and norms of reciprocity (Peters 2019). Here, social networks were characterised as structural dimensions, while subjective norms of trust and reciprocity were cognitive and/or relational dimensions (e.g. (Brown and Sonwa 2018). Structural and subjective aspects (trust and reciprocity) were often argued to be closely intertwined and mutually reinforcing in shaping outcomes (e.g. Bankoff 2007). However, most studies emphasised the structural connectivity between different types of actor more than subjective aspects (Smith and Frankenberger 2018).

The fourth conceptualisation viewed social capital as a dynamic relationship between social networks and socio-cultural dimensions. Together these were considered to shape expectations, attitudes, actions and outcomes (Wickes et al. 2017), such as willingness to cooperate and experiment, pro-environmental actions and more sustainable environmental outcomes (Kizos et al. 2014). Here, socio-cultural dimensions included values, identities, norms, beliefs and traditions that encourage or constrain actors’ actions, and resulting outcomes (Carrico et al. 2019). These socio-cultural and structural dimensions of social capital interact dynamically to shape expected and actual access to and control over different resources (Lisnyj and Dickson-Anderson 2018).

Overall, few studies considered subjective socio-cultural aspects in detail, usually focusing on outcomes for specific social groups. At the community-level studies tended to focus on trust and reciprocity. Other socio-cultural dimensions (e.g. social norms and values) were often considered superficially, without explanation about connections between multiple socio-cultural and structural dimensions (Hurlbert and Mussetta 2016). Some recent studies provide more integrative conceptualisations of social capital (e.g. Bakker et al. 2019) by, for example, emphasising social identities and norms of solidarity. However, overall the limited acknowledgement of socio-cultural dimensions may foster misleading interpretations about the type of outcomes that emerge from different social networks.

This is relevant for climate change as both mitigation and adaptation are needed across all levels of society. Thus, overlooking the role of underlying socio-cultural dimensions may place undue emphasis on structural aspects (as most studies did) that could hinder understanding how outcomes may (or may not) come about to enhance resilience to climate change.

#### Conceptualisations of the role of social capital in resilience

**Fig. 1:**
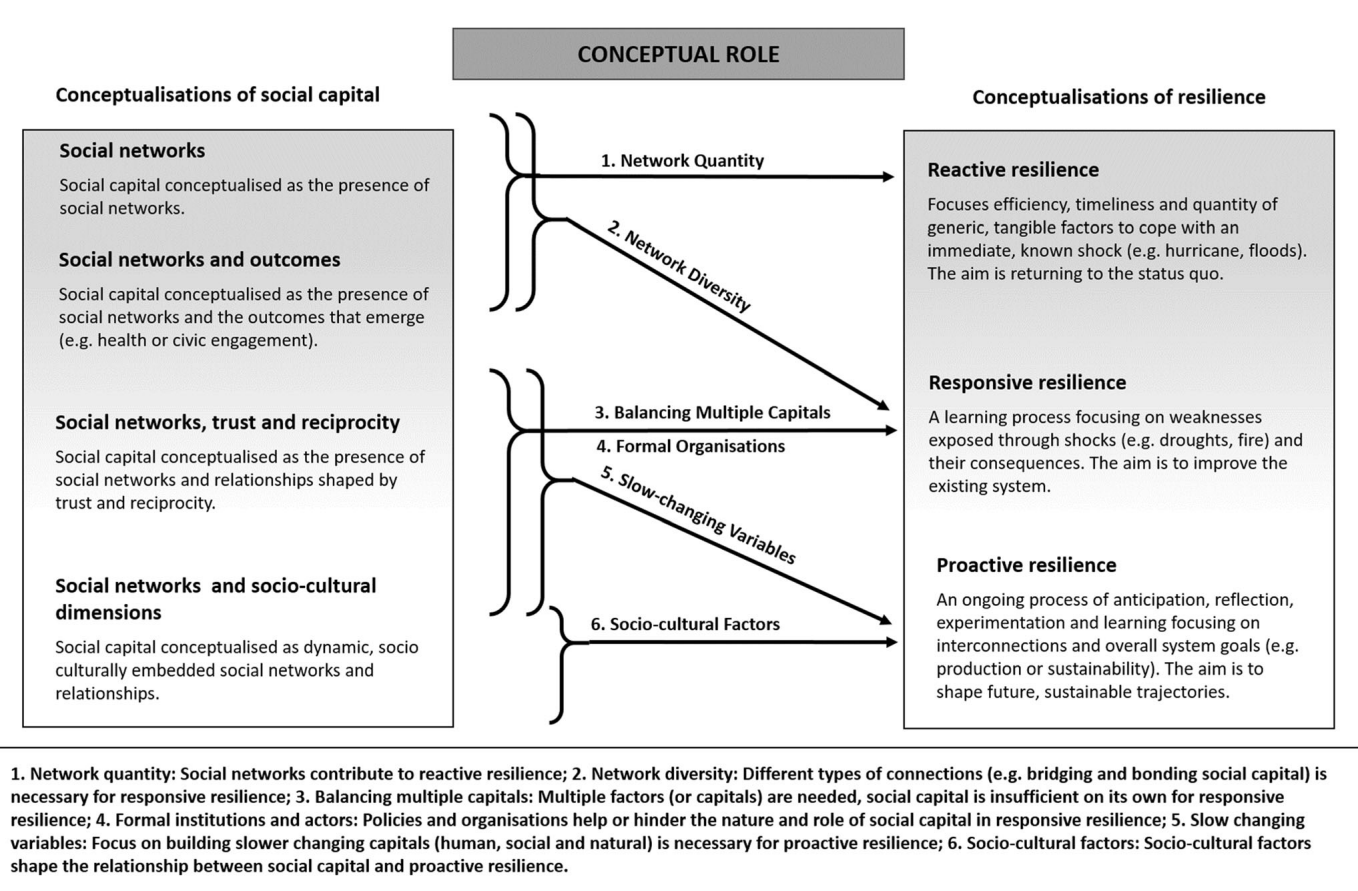
Six ways social capital was considered to influence or give rise to resilience

When the two concepts were brought together, six different conceptualisations emerged of how social capital was expected to influence or give rise to community-level resilience (Fig. 1).

The first conceptualisation related to network quantity. Here, the number of links among agents within social networks was assumed to increase social support, information and good will, which in turn was viewed as important for enhancing the ability to respond to shocks (i.e. reactive resilience) (Cassidy and Barnes 2012). Many of these studies viewed ‘bonding’ social capital (social networks of family and friends) as a key buffer to adversity (Aldrich and Meyer 2015) which needed to be cultivated before shocks (e.g. a fire) and activated when needed (Wickes et al. 2017). Such studies suggest social networks develop over time, and provide critical collective resources to minimise disruptions from climate related shocks. However, these generally focused on quantifiable aspects, and excluded consideration of more subjective dimensions shaping resilience.

The second conceptualisation of how social capital leads to resilience was through network diversity. Here, different types of social capital (e.g. both bonding and bridging) were considered important for moving beyond dealing with an immediate crisis, to also identify areas for improvement (Jordan 2015). In such studies bonding social capital was emphasised as important for coping with adverse conditions and shocks within communities (Barrett et al. 2011). Bridging social capital was considered necessary for new information, ideas, and knowledge to help shape learning, decision-making and cooperation between groups (Blackman et al. 2016), such as between communities and government agencies (Smith et al. 2012b). This conceptualisation often assumes that diverse social networks (those that enable access to existing sources of support *and* new ideas) are important for more effective responses to future climate change impacts. However, these studies did not usually consider a wide range of factors or their interactions as being important in shaping resilience.

The third conceptualisation was that social capital in the form of networks, trust and reciprocity was important for resilience—but that other assets, capacities or collective resources were also required (Singer et al. 2015). Here, a need for active management of a combination of natural, physical, economic and human factors was emphasised, but with limited overall explicit consideration given to how such factors interacted or to the wider cultural dimensions involved (Kim and Marcouiller 2016). Thus, while this conceptualisation suggests that resilience building is a multifaceted social process, studies mostly focused on how these gave rise to responsive resilience. These studies provided limited understanding of the less tangible and subjective dimensions relating to social capital and resilience.

The fourth conceptualisation emphasised the role of formal organisations and how these contribute to responsive forms of resilience. While considering social capital as networks, trust and reciprocity, such studies underscored the importance of laws, national policy, regulatory frameworks and actors (local government and non-government organisations), in helping or hindering social capital and, resilience (Hossain and Rahman 2016). Linking social capital, and the ideas and practices within formal organisations, were considered important for shaping collective action (Oteng-Ababio et al. 2015) and for identifying and making adjustments in communities for building resilience (Blackman et al. 2016). Socio-cultural dimensions relating to power and access to formal processes were sometimes considered (Jacobs and Cramer 2017), but the central focus remained on behaviours of formal actors, rather than on less visible underlying socio-cultural factors. Thus this conceptualisation suggests that the goals and practices of formal actors across levels of governance hold strong influence over effective responses to climate change impacts.

The fifth conceptualisation involves network structures, norms and trust being related to proactive resilience. These emphasised the importance of enhancing slow-changing factors (e.g. the nature of social relationships, experiential knowledge and natural resources) that would, in the long term, shape proactive resilience (Kizos et al. 2014). This viewed changes over long periods of time in natural, human, cultural and social capital as having important implications for flexibility and adaptability (Wilson 2010). This perspective emphasises that joined-up management, focused on slow-changing capitals across social scales, is important for overcoming a range of climate challenges as they emerge.

The final conceptualisation highlighted the significance of socio-cultural dimensions of social capital in shaping proactive resilience. Here the core assumption was that socio-cultural dimensions (e.g. social norms, identities and values that influence collective efficacy and agency) are central to proactive resilience processes (Skerratt 2013). These socio-cultural dimensions included subjective aspects such as sense of place, belonging, norms, identity and values and considered these as closely entwined with material aspects (e.g. place) (Cox and Perry 2011). For example, resilience could be proactively developed by overcoming collective norms that exclude or favour certain types of actions, or that promote a willingness to change (Béné et al. 2016; Smith et al. 2012b). This conceptualisation also assumed there are dynamic interconnections between multiple actors, identities and goals, and explicitly emphasised an important role for power and agency in shaping resilience (Jacobs and Cramer 2017). From this perspective, socio-cultural factors are important in shaping which aspects of climate change are recognised in decision-making and prioritised for action, which actors are involved and who benefits.

Overall these conceptualisations show the diverse ways in which social capital is considered to give rise to, or enhance, resilience, with some ways of thinking (e.g. conceptual roles 1–4 in Fig. 1) more prevalent than more nuanced understandings (e.g. conceptual roles 5 and 6 in Fig. 1). This diversity is derived from the different ways in which social capital and resilience are defined, reflecting different underlying epistemologies. For example, a focus on purely structural dimensions of social capital and on resilience to specific climate shocks (Cassidy and Barnes 2012) tended to reflect positivist perspectives. These promote a focus on finding ways to enhance resilience to immediate shocks, with less attention paid to deeper social aspects which affect disadvantage and resilience but operate over longer timeframes. In contrast conceptualisations that emphasised how diverse socio-cultural factors related to social capital were more likely to view resilience as proactive. These reflect interpretivist perspectives (e.g.Cox and Perry 2011), and place greater emphasis on the deeper underlying causes of challenges that emerge for communities. These differences are important as they greatly influenced the kinds of approaches and practice that might be adopted to enhance resilience (Moses and Knutsen 2012). For example, a focus on network quantity and diversity, with emphasis on reactive resilience, leans towards actions that focus on climate impacts and seek to help a community return to normal, rather than responding to climate change in a way that explores deeper causes. Thus, the epistemological and ontological foundations of different understandings of the role of social capital for resilience matter for research and practice. Being more explicit about such assumptions could help identify the need for broader perspectives to advance understandings, e.g. about how different connections and outcomes unfold across settings and for different types of resilience.

### How does social capital contribute to resilience?

This section turns to empirical insights from different studies about how social capital shapes resilience and identifies implications for practice. These findings are organised around three overarching themes of: (1) the role of social capital in influencing resilience; (2) factors that interact with social capital to influence resilience and; (3) the influence of formal organisations (Table 2).

**Table 2:** Empirical insights about the role of social capital in resilience and implications for community resilience and climate change practice

Theme	Empirical insights from social capital and resilience literature	Example literature	Implications for community resilience practice in the context of climate change
Role of social capital in influencing community resilience	Bonding social capital enhances reactive resilience	Murphy (2007) and Baral and Stern (2011)	Developing community resilience plans and actions that build bonding social capital is needed to help account for shocks to improve the role of social capital in enhancing reactive resilience
	Bridging (including linking) social capital contributes to responsive resilience at the community level by providing access to new resources (e.g. physical and financial) but bonding social capital shapes whether and how action is undertaken	Smith et al. (2012b), Birhanu et al. (2017), and Bakker et al. (2019)	Building bridging social capital is important to create opportunities for accessing and including diverse perspectives, new resources and ideas for decision-making, improving collective capacity for understanding and adapting to changing circumstances to enhance responsive resilience
	Perceptions of unequal access to resources can cause distrust and a loss of social capital and access to resources for future (responsive) resilience. Such losses within a community (e.g. between neighbours) may be buffered by norms of community support	Berke et al. (2008) and Islam and Walkerden (2014)	Working with less visible subjective and normative dimensions of social capital is necessary to maintain flexibility to access different types of resources over time, supporting the role of social capital for enhancing responsive community resilience in the longer term
	Social capital can facilitate learning but what is learnt, by who and whether this informs future decisions is shaped by norms of inclusion/ exclusions, thus influencing the type of resilience	Barrett et al. (2011), Wickes et al. (2017), and Baehler and Biddle (2018)	Promoting norms of inclusion within decision-making spaces is essential to develop the role of social capital for understanding different needs and perspective to shape action to enhances responsive community resilience, e.g. to understand and engage with climate disadvantage and to shape positive community narratives
Factors that interact with social capital to influence resilience	Social capital is one of many other factors involved in shaping resilience	Cassidy and Barnes (2012) and Smith et al. (2012a)	Considering the role of multiple factors and how these vary between settings is necessary when developing strategies, plans and actions for building resilience
	Social capital connects in complex ways with other slow and fast changing factors to shape resilience. Feedbacks between slow-changing factors relating to human, cultural and social capital are particularly important	Kizos et al. (2014), Sinclair et al. (2014), and Guillotreau et al. (2017)	Working through the connections between social, human and cultural factors is important to shape how desirable futures are imagined and pursued, and identify transformative need and potential to shape proactive community resilience
	Social capital is necessary but insufficient for shaping resilience, even in settings with high levels of social capital. But, social capital can be an effective strategy to develop or access hard-to-reach resources	Islam and Walkerden (2014), Jordan (2015), and Béné et al. (2016)	Creating enabling socio-political environments with diverse capacities and resources orientated towards supporting proactive community resilience is necessary to ensure a central role for social capital in building proactive community resilience in practice
	Combinations of different types of social capital and other resources will vary in importance for shaping resilience across different social settings and objectives	Smith et al. (2012b), Skerratt (2013), and Oteng-Ababio et al. (2015)	Applying social capital approaches in practice needs to focus on working with combinations of factors, which influence how problems, solutions and desirable futures are imagined and the type of spaces that emerge for new ideas, understandings (e.g. positive narratives) and outcomes to emerge (e.g. address local needs while engaging with climate action including emissions reductions)
	Social capital shifts as proximities, needs, routines and practices of actors shift, thus the role of social capital for resilience can also change over time	Vallance and Carlton (2015) , Blackman et al. (2016), Tilt and Gerkey (2016), and Peters (2019)	Finding ways for practitioners to support and strengthen bonding and bridging social capital as circumstances shift (e.g. during crises) is important for maintaining flexibility and the ability to work through vertical and horizontal connections to enhance community resilience in the longer term
	Socio-cultural factors, e.g. norms of inclusions/ exclusion, sense of community and sustainable use of shared resources, facilitate collective agency to build community resilience	Smith et al. (2012a), Parés et al. (2018), Carrico et al. (2019), and Moreno et al. (2019)	Working with social capital approaches to enhance resilience must involve engaging with the underlying socio-cultural dimensions to identify and build on opportunities and needs to guide different resilience outcomes to help give rise to proactive types of community resilience
The influence of formal institutions in shaping the role of social capital for resilience	Decisions at higher levels of governance that shift the balance of power between actors can influence different actors’ practices and social capital (structural and norms of cooperation or competition) that shape resilience	Kizos et al. (2014), Sinclair et al. (2014)	Recognising and actively supporting all types of social capital by national policy makers is important to ensure high level decisions do not undermine, and instead help strengthen vertical and horizontal connections, to enable the flexibility for community actors to enhance all types of community resilience
	Limited recognition of the importance of linking social capital can lead to missed opportunities for more coordinated collective action and further development of social capital for enhancing resilience	LaLone (2012), Morris et al. (2019), and Thompson and Lopez Barrera (2019)	Working through vertical connections is important to ensure local government interventions are designed to connect with local needs and capacities and build all types of social capital in implementation, enhancing the role of social capital in promoting resilience in the long term
	Linking social capital can help create new opportunities to enhance social capital, e.g. through the creation of voluntary and transformational leadership programmes to enhance community resilience	Madsen and O'Mullan (2014) and Webb et al. (2016)	Building and working through linking social capital helps create opportunities for developing and strengthening government supported interventions, including those aimed at enhancing the role of social capital to support resilience within communities. Enhance proactive community resilience however needs to involve opportunities for meaningful participation in decision-making, collective learning and for empowering forms of change
	Embedded institutional socio-cultural factors (discourses, attitudes and practices) can influence the access of social groups to different spaces and resources that shape resilience	Cox and Perry (2011), Oteng-Ababio et al. (2015), and Singer et al. (2015)	Engaging with and shaping government cultures, values and practices of these actors is critical to strengthening enabling policy environments to develop the role of social capital in building community resilience,, particularly for engaging with complex challenges including climate change

#### The role of social capital in influencing resilience

There were four key findings about how social capital influences resilience. First, the ability of households to cope during crises was enhanced by bonding capital, such as in the immediate aftermath of floods, cyclones, fires and during more prolonged crises such as droughts. Bonding social capital enhanced access to psychological and material support (Birhanu et al. 2017) and operated as a strategy for households to cope more effectively with crises (Béné et al. 2016) and while directly contributing to reactive resilience, this is important for all forms of resilience building..

Bridging and linking social capital were also important in the immediate aftermath of crises (e.g. within a few days post-flood) for enhancing access to new information, resources and support to address immediate and future material losses, e.g. access to building materials and financial aid (Birhanu et al. 2017). Bonding combined with limited bridging social capital, however, was shown to limit whether, and how, a need for change is perceived and acted upon (Bakker et al. 2019), such as collectively recognising climate change as a threat but with factionalised views on the type of action and change required (Smith et al. 2012b). Thus the collective learning needed to improve responsive resilience can be helped or hindered through different combinations of social capital.

Tensions around the distribution of resources in the immediate aftermath of crisis led to a longer-term loss of bridging and linking social capital. For example, community relationships between households (and to aid organisations) were weakened from competition for accessing scarce external support (Islam and Walkerden 2014). Existing norms that emphasised community support fostered the development of social capital prior to crises, while also reducing tensions during crises, thus preventing potential losses of social capital in the future once a crisis had abated (Berke et al. 2008). Thus, underlying socio-cultural norms focused around community support and cohesion may support resilience building over time, by both encouraging the development of social capital and buffering against potential losses from conflict as communities move through periods of scarcity.

Finally, crises created space for learning about how to strengthen a local settings to reduce future negative consequences (Wickes et al. 2017). Specific crises can contributed to responsive resilience as the community learnt and updated its understanding of issues and factors hindering resilience generally. Critical aspects affecting whether learning contributes to responsive resilience were the distribution of learning (e.g. the different actors involved and the flow of ideas and knowledge), the type of learning (e.g. understanding weaknesses in physical infrastructure and/or the need for coproduction approaches to build resilience), and whether and how this learning informs collective decision-making (Blackman et al. 2016). Social capital, however, can also limit learning and decision-making if it excludes different perspectives and learning practices (Brown and Sonwa 2018). Thus although social capital can support experiential learning, the type of learning that emerges and for who, and thus the type of resilience that unfolds, varies across contexts.

These results show that social capital has an important yet dynamic role in resilience framed in terms of shocks and crises (e.g. floods and droughts). This role is often explained in terms of more visible structural dimensions of social capital (the quality and diversity of bridging and linking social capital) and the distribution of resources and learning. However, less visible dimensions (e.g. perceptions and social norms) are also important to shape structural dimensions of social capital and for learning and accessing resources. For example, social norms can lead to trade-offs between structural types of social capital, can mediate tensions and their impact, and how learning informs action. Thus social network structures interact with less visible, underlying social norms to help shape the type of resilience that emerges. For resilience and climate change practice working with structural aspects of social capital is important for overcoming climate shocks. To build proactive resilience and engage with the many aspects of climate change and future uncertainties involved, there is a need to work with less visible dimensions within communities. This is important to support social capital in ways that shape action suited to diverse settings and actors, and to enhance flexibility for the future.

#### Factors interacting with social capital to influence resilience

There were a six key findings about different factors that interacted with social capital to influence resilience. First, while social networks were widely found to be a key resource for times of change (Tilt and Gerkey 2016), a range of other factors were important, including natural resources, livelihoods, knowledge and experience, the built environment and financial resources that together shaped decisions and actions for resilience (Baral and Stern 2011; Jordan 2015). Studies often emphasised different combinations of factors, such as the importance of natural capital in rural settings and physical capital in more urban settings. This reflects differentiation of potential resources across settings at the community level and highlights a need to consider the role of multiple factors for shaping community resilience.

Second, social capital interacted in complex ways through feedbacks with other social, human, cultural and natural factors to shape goals and practices over temporal (years and decades) and spatial scales (e.g. households to industries) (Guillotreau et al. 2017). In these studies, faster changing factors were suggested to have, overall, much less importance than slower changing factors, even for reactive and responsive resilience (Sinclair et al. 2014). Furthermore, social capital was deliberately developed by community actors to strengthen other factors for proactively enhancing resilience (Skerratt 2013). This highlights the importance of considering slow-changing factors (e.g. social capital and cultural dimensions) in shaping different types of resilience over longer timeframes.

Empirical studies also suggested that social capital is important but insufficient in shaping resilience for those who are marginalised, excluded or in contexts of high social inequality. Here, other influential factors constrained opportunities for resilience, such as in systems where bribery is common, thus hindering access to resources for some (Islam and Walkerden 2014). In such circumstances, no matter how much social capital is available, there was limited possibility for building proactive resilience (Hossain and Rahman 2016). When resources within or outside communities were available but difficult to access, as social capital helped gain access to new opportunities and resources (e.g. micro-credit) and thus helped enhance resilience (Jordan 2015). The key point here for building resilience is that although social capital may be central for shaping action, the type of outcomes that unfold are also shaped by the availability of other resources.

Fourth, different combinations of social capital, such as bonding, bridging and linking, were also found to be important for achieving different objectives (Skerratt 2013). For example, a combination of high bonding, bridging and linking social capital was found to be important for expressions of autonomy at the community level, whereas bonding capital was important for consolidating community identity (Smith et al. 2012b). Different social networks within communities, the connections between them, and the multi-functionality of these networks provided flexibility through time to mobilise different resources in relation to a range of events, from natural hazards to infrastructure failure (Murphy 2007; Vallance and Carlton 2015). The implication for community resilience practice is that collective goals and visions and how they are pursued will vary, and this is in-part shaped by different configurations of social capital.

Fifth, social capital can change over time short time frames as actors’ proximity, needs, routines and practices shifted, e.g. as crises unfolded and as neighbours were dispersed (Singer et al. 2015). In social settings with limited resources this led to a loss of resilience in the long term (Tilt and Gerkey 2016). Such disruption also created opportunities to form new relationships and shape new shared initiatives to meet arising needs and to spread ideas and information for enhancing responsive resilience (Vallance and Carlton 2015). This highlights that although pre-crisis social capital is important for resilience to shocks, the disruption involved can alter social capital in the long term.

Finally, socio-cultural factors associated with social capital played a substantial role in shaping resilience. Norms, values and identities influenced the form and function of networks, such as exclusionary norms that lead to isolated factions, hindering the development of bridging social capital or norms that perpetuated unsustainable practices (Carrico et al. 2019). Cultural norms that contribute to collective agency related to good neighbourliness, solidarity and activism (Sinclair et al. 2014; Parés et al. 2018). Community-level socio-cultural factors were also identified as important in shaping how social capital was applied in enhancing resilience, e.g. shaping acceptance of the status quo and thus the ability to collectively imagine an alternative future (Birhanu et al. 2017). Specific factors (or aspects of communities) that had particular symbolic value for community identity were also shown as important for collective agency (Smith et al. 2012a) and if social capital was actively pursued as a resilience building strategy (Skerratt 2013). Thus socio-cultural dimensions within communities shape connections between agency, social capital and resilience. For practice this implies the need to work with socio-cultural dimensions within social capital to guide how community resilience unfolds.

These results show social capital is one of many factors that dynamically interact to shape resilience. Social capital can be actively used to access hard-to-reach resources and shape other aspects of communities. However, for resilience building over longer timeframes, interconnections between slower changing factors are particularly important. These include social and human capital, and underlying socio-cultural factors – such as values, social norms and collective identities that shape overarching goals, perceived resources and collective agency. Thus, systems perspectives and the active engagement of multiple, interconnected factors, including social capital and the underlying socio-cultural factors involved, need to incorporated into community resilience building strategies. With the complexities of climate change proactive types of resilience practice needs to account for multiple interacting factors and scales while maintaining flexibility for the future. Such factors influence collective visions, goals and perceived needs at the community level for building resilience, (e.g. if adapting to and mitigating climate change are both considered), the relevance of social capital for shaping actions (e.g. the type and quality of social capital), what emerges and for whom (e.g. group or community scale). The ability to guide and work with social capital in combination with other factors is important to avoid overly simplistic approaches to social capital and resilience. In terms of climate change this is important for applying systemic approaches in practice, consideration of different challenges and for building inclusive, positive climate narratives to strengthen resilience practice.

#### The influence of formal organisations on the role of social capital for resilience

Four key findings were also identified around the influence of formal organisations in shaping the role of social capital for resilience. This included a focus on ideas, decisions and actions of different organisations, and national and local level programmes and government policies.

First, decisions at higher levels of government were found to shape local decisions and practices that reduced social capital and resilience (Luthe and Wyss 2015). For example by altering power dynamics between actors and changing the way they interacted, bridging and bonding social capital was eroded (Kizos et al. 2014). This loss of community resilience occured through ideological shifts in national-level policy processes, e.g. towards market-based approaches that increase competition between local producers (favouring individualism over cooperation) or towards technical rather than holistic solutions (Sinclair et al. 2014; Guillotreau et al. 2017). For community resilience, the role of social capital can be unintentionally eroded overtime through government change programmes.

Second, limited linking social capital between local organisations and communities led to missed opportunities for coordinating different resources (e.g. in response to a crisis or shock). This lack of social capital can cause a mismatch between actions of communities and local organisations (LaLone 2012). Better coordination can emerge from regular interactions between actors and improve the quality of social capital for the future (Thompson and Lopez Barrera 2019). This highlights that social capital is a dynamic resource that can be strengthened when activated over time to enhance resilience.

Third, some formal organisations (e.g. state agencies and non-government organisations) provided support via funded programmes and linking social capital. These are important for enhancing community resilience in direct (e.g. providing access to micro-credit) or indirect (development of transformational leadership skills) ways, that in turn enhanced social capital (Madsen and O'Mullan 2014). Here, the presence of linking social capital between formal organisations and communities shaped programme outcomes, such as increasing access to critical financial support and indirectly supporting the development of social capital within communities. This highlights that formal institutions can have a role in strengthening social capital for building community resilience, however in practice the effectiveness of such interventions is shaped by linking types of social capital.

Finally, socio-cultural dimensions of relations between communities and local organisations were suggested to shape community resilience indirectly. For example, perceptions of injustice in the practices of formal organisations (e.g. distributing resources) may indirectly hindered social capital by exacerbating tensions between community-level actors (i.e. between neighbours) (Tilt and Gerkey 2016). Formal organisations with top-down leadership approaches often lacked a social capital mind-set that may not create sufficient space for communities to lead decision-making to understand and address current and future needs for improving responsive resilience (Blackman et al. 2016). Furthermore, practices with formal organisations that overlooked the role of social capital led to indirect, unintended losses of social capital and limited opportunities to enhance community resilience (Cox and Perry 2011). This suggests practices and norms within formal organisations are important for shaping community resilience and over time, the role of social capital in these processes.

These findings show that formal organisations are important actors for shaping the nature and role of social capital for community resilience. At a national level, policy paradigm shifts may alter the nature of social capital and thus the accessibility of resources for different actors. At a community level, the behaviour, attitudes and actions of organisational actors may directly and indirectly influence the nature of social capital and its role in resilience building. For resilience and climate change practice this emphasises the need or enabling policy environments for strengthening the role of social capital and for shaping resilience approaches more broadly. To better enable proactive resilience building at the community level, such policy environments need to help bring diverse perspectives, ideas and capacities together and adopt a social capital approach to create spaces for exploring, learning and synergistic actions.

### Critical knowledge gaps for studies of resilience, social capital and climate change

The previous sections examined conceptualisations (Fig. 1) and empirical insights (Table 2) about the role of social capital for resilience, drawing out insights for community resilience and climate change practice. This section identifies four critical knowledge gaps to advance understandings on this subject for researchers and practitioners.

#### Why and how outcomes emerge through social capital, not just what emerges

Across this diverse body of literature understandings of the role of social capital are often framed in terms of type of social capital, e.g. bonding, bridging and/or linking social capital or strong and weak ties that connect different types of actors and in terms of outcomes such as learning and access to resources. This strong orientation towards structural understandings of the role of social capital (i.e. network quantity and diversity) however provides limited insights to understand *how* and *why* different outcomes unfold. Empirical findings suggest that the binary existence of social relationships and networks (i.e. whether or not agents are connected) appears to be less important than the nature of those relationships in shaping what emerges for enhancing resilience. Improving understanding about why and how different outcomes unfold (and for whom) through social relationships requires greater attention to a wide diversity of factors that influence the nature (i.e. type and quality) of social capital, as well as different perspectives and goals that also influence decisions that shape actions and types of resilience. Addressing this gap is important to better understand how to guide social capital approaches in practice for engaging with complex challenges such as climate change in ways that support proactive resilience building at the community level.

#### Dynamic interactions between different factors and social capital over time

Different factors, resources and/ or capacities are emphasised within conceptual understandings of resilience alongside social capital. Conceptually this includes a focus on the importance of slow-changing capitals (i.e. human, natural, cultural and social capital that changes over decades) for shaping resilience. Empirical studies have tended to focus on tangible factors, e.g. infrastructure and indicators of economic development in the emergency planning literature, with an emphasis on reactive resilience. There is limited empirically understanding about how multiple factors dynamically connect and influence each other across levels to reinforce or dampen resilience capacities. Better understanding of the dynamic relationships between social capital and other diverse factors is particularly important for resilience that explicitly recognises the need for change (i.e. responsive and proactive resilience processes), the systemic nature of climate change and the potential for factors to combine in ways that may enhance or weaken community resilience through time. This is important to develop understanding of resilience as a complex social process, and how to nurture it in practice.

#### Different ways formal organisations can shape social capital and community resilience

Formal organisations (e.g. national and local government) are often emphasised, both conceptually and empirically, as important in shaping the role of social capital for community resilience. Currently studies tend to adopt top-down, hierarchical perspectives that assume formal organisations and policies direct resilience building, and often focus on reinforcing the status quo (i.e. reactive and responsive resilience). This may hinder engagement at the community level with the complexities of climate change for proactive forms of resilience to emerge. By ignoring questions of unequal power relations, opportunities are missed to improve understanding about the different ways formal organisations could potentially support social capital and resilience in communities. Alternative perspectives on approaches within formal organisations are rare, particularly for supporting proactive resilience at the community level. Such perspectives may involve examining how formal organisations can ‘flatten’ hierarchies by altering the dynamics between actors, and creating space for the co-development of locally relevant resources and actions for resilience. This may involve a subtle shift in focus towards shaping change within formal organisations to enhance social capital approaches in practice for proactively building community resilience in the context of climate change.

#### Socio-cultural dimensions shaping the nature and role of social capital for community resilience

Socio-cultural factors are often conceptually underplayed. When these factors are considered this is often within studies that examine social capital in relation to proactive resilience processes, such as how values and norms influence foresight, reflection, experimentation and learning, and hence resilience. This includes a particular focus on social norms of exclusion in limiting the role of social capital in resilience for marginalised social groups. Empirical aspects of studies however highlight the role that less visible socio-cultural dimensions (e.g. values, norms and beliefs) play in social capital within different community resilience processes, such as norms of community support. Such dimensions shape meanings attached to ideas, goals, resources and interactions between actors and thus in part influence the type of outcomes and who benefits. Yet such socio-cultural considerations are not common, especially in studies focusing on resilience at the community level. However, the need to consider such factors in change processes and engage with social justice challenges is increasingly recognised. Improving conceptual recognition and empirical knowledge on how socio-cultural dimensions shape community resilience processes (including those shaping connections between climate action and marginalisation), and the nature and role of structural aspects of social capital in these processes is therefore critical for understanding how different local needs and aspects of climate change are incorporated into resilience building processes.

Addressing these knowledge gaps will involves interpretivist perspectives to build on the positivist ways of thinking about social capital and resilience that currently dominate. This is important for more nuanced understandings about the role of social capital and how to guide community-level resilience building that engages with the complexities of challenges like climate change. Better understanding how the different dimensions of social capital (both structural and socio-cultural dimensions) interact to shape the form of and different outcomes from social networks and relationships, and how social capital interconnects with other factors within community resilience building processes are important for informing practice. However, without intervention that explicitly engages with multiple, interconnected challenges to enhance resilience, the potential for erosion of community resilience over time is much greater. Thus greater attention must be paid to proactive forms of resilience, its socio-cultural aspects, and how to work dynamically through social capital and with other factors to strengthen approaches goal and actions to guide this in practice.

## Conclusion

This review synthesised conceptual and empirical understandings of the role of social capital in resilience, and implications for community resilience and climate change practice. Multiple conceptualisations of resilience, social capital and its role are highlighted. While resilience was often framed in terms of shocks (e.g. climate change impacts) and maintaining the status quo, social capital often involved a strong emphasis on structural dimensions, with socio-cultural elements underplayed. This influenced how the role of social capital in resilience was understood, with a strong emphasis on the quantity and diversity of (structural) social capital, other factors alongside social capital, and the influence of formal organisations. Empirical findings therefore reiterate the importance of social capital for community resilience, while showing the complex ways they can interact. The many nuances in empirical findings, such as potential for certain forms of social capital to constrain community resilience, suggest underlying socio-cultural factors are particularly key. They shape structural dimensions of social capital (the type of actors involved) and what emerges (the type of outcomes and for whom) to contribute to different types of resilience.

Social capital approaches therefore provide important leverage for catalysing action to build resilience. However, resilience and climate change research needs to go beyond simplistic, structural accounts of social capital to focus more on socio-cultural factors and how different factors interact across social levels and over time to shape different approaches, actions and outcomes. This is important for enabling more systemic, proactive approaches to community resilience to fully engage with complex interconnected climate change challenges.

## Footnotes

1 The term resilience is used to refer to resilience at any social level, and community resilience when referring specifically to the resilience of communities.

2 We follow Gibson et al. (2000, p. 218) in differentiating between scales (‘the spatial, temporal, quantitative, or analytical dimensions used to measure and study any phenomenon’) and levels, the units of analysis located on a scale.
